# Influence of the Structural Features of Carrageenans from Red Algae of the Far Eastern Seas on Their Antiviral Properties

**DOI:** 10.3390/md20010060

**Published:** 2022-01-08

**Authors:** Natalia V. Krylova, Anna O. Kravchenko, Olga V. Iunikhina, Anastasia B. Pott, Galina N. Likhatskaya, Aleksandra V. Volod’ko, Tatyana S. Zaporozhets, Mikhail Y. Shchelkanov, Irina M. Yermak

**Affiliations:** 1 G.P. Somov Institute of Epidemiology and Microbiology, Rospotrebnadzor, 690087 Vladivostok, Russia; olga_iun@inbox.ru (O.V.I.); pott_a.b@mail.ru (A.B.P.); niiem_vl@mail.ru (T.S.Z.); adorob@mail.ru (M.Y.S.); 2 G.B. Elyakov Pacific Institute of Bioorganic Chemistry, Far-Eastern Branch of the Russian Academy of Sciences, 690022 Vladivostok, Russia; kravchenko_89@mail.ru (A.O.K.); galin56@mail.ru (G.N.L.); morskaia@list.ru (A.V.V.)

**Keywords:** carrageenan, herpes simplex virus type 1, enterovirus, molecular docking

## Abstract

The structural diversity and unique physicochemical properties of sulphated polysaccharides of red algae carrageenans (CRGs), to a great extent, determine the wide range of their antiviral properties. This work aimed to compare the antiviral activities of different structural types of CRGs: against herpes simplex virus type 1 (HSV-1) and enterovirus (ECHO-1). We found that CRGs significantly increased the resistance of Vero cells to virus infection (preventive effect), directly affected virus particles (virucidal effect), inhibited the attachment and penetration of virus to cells, and were more effective against HSV-1. CRG1 showed the highest virucidal effect on HSV-1 particles with a selective index (SI) of 100. CRG2 exhibited the highest antiviral activity by inhibiting HSV-1 and ECHO-1 plaque formation, with a SI of 110 and 59, respectively, when it was added before virus infection. CRG2 also significantly reduced the attachment of HSV-1 and ECHO-1 to cells compared to other CRGs. It was shown by molecular docking that tetrasaccharides—CRGs are able to bind with the HSV-1 surface glycoprotein, gD, to prevent virus–cell interactions. The revealed differences in the effect of CRGs on different stages of the lifecycle of the viruses are apparently related to the structural features of the investigated compounds.

## 1. Introduction

Marine algae are a source of natural products with many pharmacological applications, including substances inhibiting viral infection and/or replication. Since the finding of antiviral activities in many species of marine algae and the isolation of some active compounds from them, marine algae have become recognized as potential sources of antiviral substances [1]. Red and brown algae produce sulphated polysaccharides, which, as numerous studies have shown, present a wide spectrum of antiviral activity, both in vitro and in vivo [2,3,4,5,6]. The modes of antiviral action of most sulphated seaweed polysaccharides have often been attributed to a blockade of the early stages of the virus replication cycle [7]. It is believed that the formation of specific supramolecular complexes of sulphated polysaccharide with the virus or the target cell is the basis of their antiviral activity. These complexes rely on non-covalent interactions (mainly electrostatic, but also hydrophobic and polar) and are commonly attributed to the structural characteristics and composition of the sulphated polysaccharide. Their degree of sulfation, molecular weight, and structural features are key factors in their antiviral activity, as highlighted by the chemical investigation of polysaccharides [8,9]. Among sulphated marine polysaccharides, carrageenans (CRGs) occupy a special place due not only to their chemical structure but also to their special physicochemical properties, which, in general, determine a wide range of their antiviral properties [5,6].

Carrageenans (CRGs) are a family of water-soluble linear sulphated galactans extracted from red algae belonging to the order Gigartinales. They are composed of alternating 3-linked β-D-galactopyranose (G-units) and 4-linked α-D-galactopyranose (D-units) or 4-linked 3,6-anhydro-α-D-galactopyranose (DA-units), forming the disaccharide repeating unit of CRGs [10]. Several types of these polysaccharides are identified on the basis of the structure of the disaccharide repeating units, by the sulphation pattern, and by the presence of 3,6-anhydrogalactose (A unit). The most common types of CRGs are named κ-, ι-, and λ-CRGs based on the structure of the major disaccharide repeating units, but natural CRGs are often hybrids of more than one of these units [11]. CRGs are appreciated for their structural diversity, which is associated with a large panel of physico-chemical properties and biological activities. The peculiarities of the structural types of CRGs and their molecular weight determine the biological activity of these polysaccharides [8,11].

In vitro and animal studies have shown that CRGs inhibit different enveloped RNA or DNA viruses, including herpesviruses (HSV) [12,13,14,15,16], human immunodeficiency virus (HIV) [17,18], influenza viruses (IAV) [19,20,21], dengue virus type 2 (DENV-2) [22], metapneumovirus [23], rabies virus (RABV) [24] and other viruses. The investigations of the last two years have demonstrated a pronounced activity of CRGs against SARS-CoV-2 [6,25,26]. However, only a few studies have demonstrated the ability of CRGs to effectively neutralize non-enveloped viruses, such as enterovirus 71 (EV-71) [27], human papillomavirus (HPV) [28], human rhinovirus (HRV) [29,30], and adenovirus 40 [31,32]. It should be noted that the antiviral properties of CRGs depend on both the methods of their extraction from various algae and on the structural type of the polysaccharide, the degree of its sulfation, and its molecular weight [6,11,33]. We have previously shown that CRGs obtained from the algae *Chondrus armatus* and *Tichocarpus crinitus* have different antiviral activities against the leaves of the tobacco mosaic virus (TMV) [34]. The antiviral activities of CRGs were higher than those of oligosaccharides [35].

The aim of this study was to perform a comparative analysis of the antiviral activities of different types of CRGs isolated from red seaweed families of Gigartinaceae, Tichocarpaceae, and Phyllophoraceae collected on the Pacific coast against enveloped DNA herpes simplex virus type 1 (HSV-1) and non-enveloped RNA enterovirus (ECHO-1) viruses. Investigation of the antiviral activity of CRGs and oligosaccharide can give us the answer of which structural type of these unique polysaccharides is important against viruses.

## 2. Results

### 2.1. Characteristics of CRGs

The CRGs were extracted from red seaweed *C. armatus*, *T. crinitus*, and *A. flabelliformis* purified from low molecular weight impurities by column filtration and precipitated with alcohol. The yield of total sulphated polysaccharide for *C. armatus* (designated as unfractionated or the total polysaccharide CRG1) was 33%, the total yield from *T. crinitus* was 30%, and the total yield from *A. flabelliformis* was 25%. Total polysaccharides were separated using 4% KCl or CaCl_2_ into insoluble (gelling) and soluble (non-gelling) fractions, as described in the methods section. In our work, gelling fractions of polysaccharides (CRG 2 and CRG3) and the total polysaccharide CRG1 from *C. armatus* were used.

According to chemical analysis (Material and Method), the main components of the polysaccharides were galactose, 3,6-anhydrogalactose and the sulphate groups (Table 1).

The structures of the gelling polysaccharides were studied by FTIR spectroscopy and ^13^C NMR. We compared absorption bands in the IR spectra and chemical shifts in the NMR spectra with corresponding signals of known CRG structures [36,37]. Moreover, the obtained spectra were compared with the spectra of the polysaccharides isolated by us from *C. armatus*, *T. crinitus*, and *A. flabelliformis species* [38,39,40].

**Figure 1:**
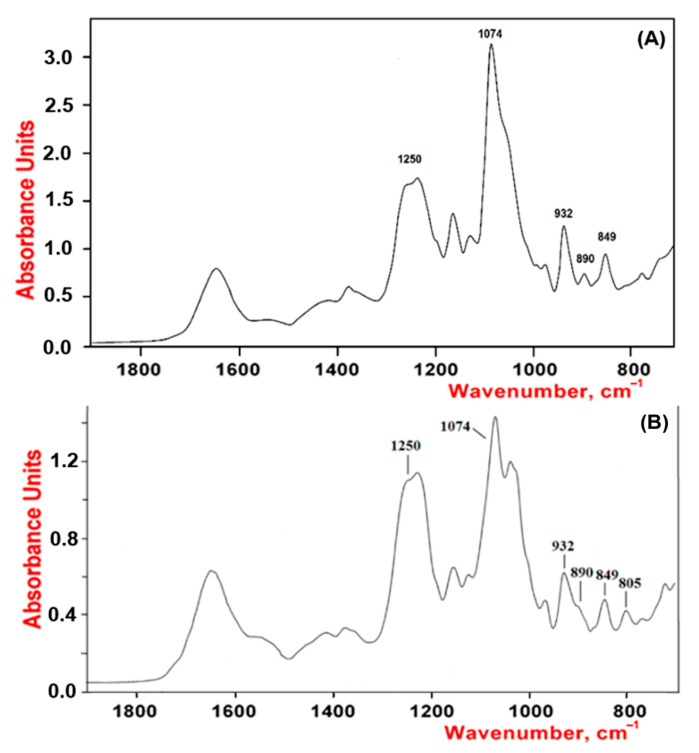
IR spectra of κ/β-CRGs from *T. crinitus* (**A**) and ι/κ-CRGs from *A. flabelliformis* (**B**).

As shown by our results, the studied polysaccharides were CRGs and had hybrid structures. In the IR spectra (Figure 1) of gelling polysaccharides from *T. crinitus* and *A. flabelliformis* absorption band in the region of 1250 cm^−1^ indicated the presence of sulfate groups (–S=O asymmetric vibration), with significant number in the case of polysaccharide from *A. flabelliformis*, that is consistent with results of chemical analysis. Absorption bands at 932 and 849 cm^−1^ in IR spectra of polysaccharides were characteristic of 3,6-anhydrogalactose (C–O vibration) and the secondary axial sulphate group at C-4 of the 3-linked β-D-galactose residue, respectively. This made it possible to assign the polysaccharides to κ-type of CRG. Moreover, the IR spectrum of the gelling polysaccharide of *A. flabelliformis* also had a pronounced absorption band at 805 cm^−1^, belonging to the secondary axial sulfate group at C-2 of a 4-linked 3,6-anhydro-α-D-galactose of ι-disaccharide unit, whereas the absorption band at 890 cm^−1^ in the IR spectrum of the polysaccharide of *T. crinitus* showed the presence of non-sulphated β-D-galactose residues, typical for β-CRG. FTIR spectroscopy data were confirmed by NMR spectroscopy (^1^H and ^13^C NMR spectra in Supplementary). In the ^13^C NMR spectrum of polysaccharide from *T. crinitus* were poorly resolved signals at 103.1 and 102.7 ppm that is the result of overlapping of the C-1 signals of the 3-linked β-D-galactose 4-sulfate of the κ-CRG (G4S) and the 3-linked β-D-galactose (G) of β-CRG. The signal at 95.1 ppm and less intense signal at 94.4 ppm in the anomeric carbon resonance region of the ^13^C NMR spectrum of *T. crinitus* polysaccharide were characteristic of C-1 of the 4-linked 3,6-anhydro-α-D-galactose of κ-CRG (DA) and β-CRG (DA’), respectively (Supplementary Table S1). Segments of κ- and β-carrabiose units were estimated by ^1^H NMR by integrating the α-anomeric signals that allowed estimation the ratio of κ- to β-units: 70:30 (Supplementary Figure S1).

The signal at 92.9 ppm in the ^13^C NMR spectrum of *A. flabelliformis* polysaccharide was assigned to C-1 of 4-linked 3,6-anhydro-α-D-galactose 2-sulfate (DA2S) of ι-CRG, and signal at 96.2 was characteristic of the C-1 of 4-linked 3,6-anhydro-α-D-galactose of κ-CRG (DA). Poorly resolved signals at 102.9 and 103.1 ppm were the result of overlapping of C-1 signals of 3-linked β-D-galactose 4-sulfate of ι-(G4S) and κ-CRGs (G4S), respectively. The ^13^C NMR spectrum in the upfield region was typical for ι- and κ-types of CRGs (Supplementary Figure S2). The ^1^H NMR spectrum corroborated ^13^C NMR spectroscopy data. Having integrated the signals of anomeric protons at 5.09 and 5.30 ppm corresponded to 4-linked 3,6-anhydro-α-D-galactose of κ-CRG (DA) and 4-linked 3,6-anhydro-α-D-galactose 2-sulfate of ι-CRG (DA2S), respectively. In addition, the signal with a weak integrated intensity at 5.50 ppm in the ^1^H NMR spectrum was assigned to the H-1 of α-galactose 2,6-disulfate of ν-CRG (the biosynthetic precursor of ι-CRG).

Thus, the presented polysaccharides isolated from different algae species characterized by the presence of 3,6-anhydro-D-galactose had hybrid CRG-structure but differed in the number of repeating units of CRG and the number of sulphated groups. Thus, along with the κ-carrabiose units, the polymeric chain of CRG from *T. crinitus* contained non-sulphated β-carrabiose units, while CRG from *A. flabelliformis* contained strongly sulphated ι-carrabiose units. Thus, these polysaccharides have been identified as κ/β-CRG from *T. crinitus*, with a ratio of κ- to β-units of 70:30 [41] and ι/κ-CRG from *A. flabelliformis* [39]. Along with them, to study activity unfractionated Σ-CRG was used, which consists of κ- and λ-CRGs with a ratio of 60:40, as was shown earlier [38], and oligosaccharides of ι/κ-CRG (ι/κ-CRG-OS) from *A. flabelliformis*.

Oligosaccharides of ι/κ-CRG (ι/κ-CRG-OS) were produced by mild acid hydrolysis of the native polysaccharide, which does not result in the destruction of 3,6-anhydrogalactose. The conditions of hydrolysis were selected by us earlier [42]. According to the chemical analysis, the main components of ι/κ-CRG-OS as the ι/κ-CRG were galactose, 3,6-anhydrogalactose, and sulphate groups (Table 1).

**Figure 2:**
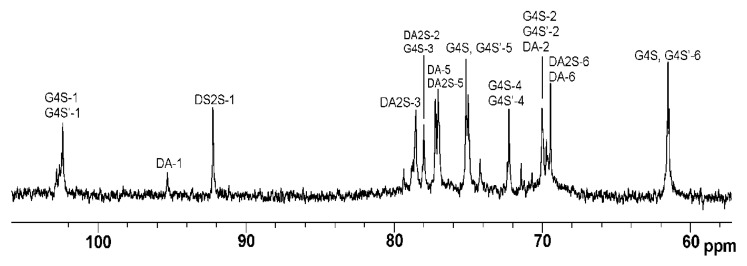
^13^C NMR spectrum of oligosaccharides of ι/κ-CRG (ι/κ-CRG-OS).

The ^1^H and ^13^C NMR spectra of ι/κ-CRG-OS obtained by mild acid hydrolysis at 37 °C for 44 h were identical (Figure 2) to the spectra of the original polymer, except that the oligosaccharides contained even more ι-units and the ratio of ι- to κ-CRG was 85:15.

### 2.2. Cytotoxicity and Antiviral Activity of CRGs

**Figure 3:**
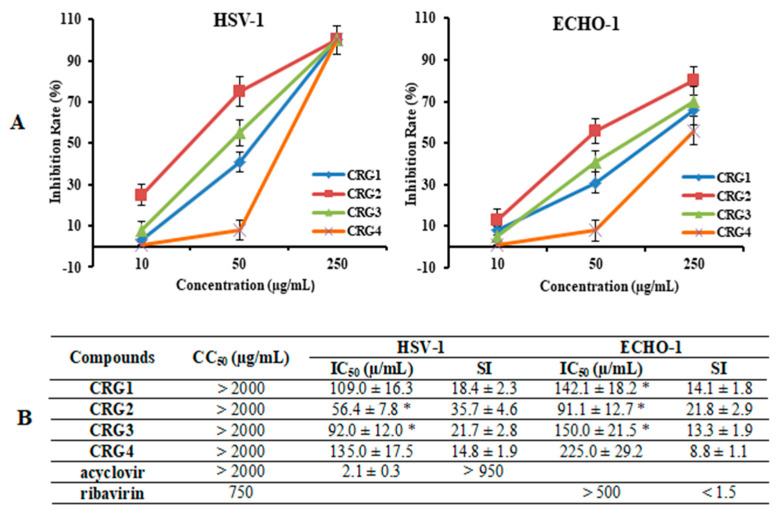
Inhibitory effect of CRGs against herpes simplex virus type 1 (HSV-1) and enterovirus (ECHO-1). Vero cells were infected with HSV-1, ECHO-1 (100 PFU/mL) and simultaneously treated with several concentrations of tested compounds. The antiviral activity of compounds was determined by plaque reduction assay and values are expressed as means ± SD of three experiments, each performed in triplicate. (**A**) The dose-dependent antiviral activity of CRGs (CRGs) against HSV-1 and ECHO-1 and data represented the rate of inhibition of the plaques (%). The inhibitory concentration (IC50) values of the CRGs were calculated by regression analysis of the dose–response curves; (**B**) Lists the IC50, cytotoxic concentration (CC50), and selectivity indices (CC50/IC50, SI) values. * Significance of the differences between the parameters of CRG polysaccharides (CRG1, CRG2 and CRG3) compared to CRG oligosaccharide (CRG4) (*p* ≤ 0.05).

The cytotoxicity assessment of the tested CRGs (CRG1, CRG2, CRG3 and CRG4) and reference compounds (acyclovir and ribavirin) against Vero cells was carried out by means of the methylthiazolyltetrazolium bromide (MTT) assay. The studied compounds showed low toxicity of all the CRGs and acyclovir to Vero cells: their 50% cytotoxic concentrations (CC_50_) were above 2000 µg/mL, while the CC_50_ of ribavirin was 750 µg/mL (Figure 3B). An additional antiviral activity assay was performed for compounds with concentrations below 500 µg/mL.

The anti-HSV-1 and anti-ECHO-1 activities of the investigated compounds were assessed using a plaque reduction assay. Vero cells infected with 100 PFU/mL of the corresponding virus were simultaneously treated with different concentrations of compounds. The dose-response curves shown in Figure 3A demonstrated that CRGs inhibited virus-induced plaque formation in a dose-dependent manner, and the inhibitory concentrations (IC_50_) of CRGs for both viruses were calculated using regression analysis of these curves (Figure 3B). Polysaccharides CRG1 and CRG3 revealed moderate antiviral activities against HSV-1 and ECHO-1, whereas CRGs oligosaccharide showed weak activities.

At the same time, the most active sample toward both viruses was CRG2, the antiviral effect of which was two times higher than that of CRG4 (*p* ≤ 0.05). Furthermore, based on the IC_50_ and SI values, all investigated CRGs more effectively inhibited plaque formation of the enveloped virus, HSV-1, than the non-enveloped virus, ECHO-1. Meanwhile, acyclovir showed the highest antiherpetic activity (SI > 950), and ribavirin showed the lowest anti-enteroviral activity (1.5).

### 2.3. Antiviral Mode of Action of Investigated CRGs

**Figure 4:**
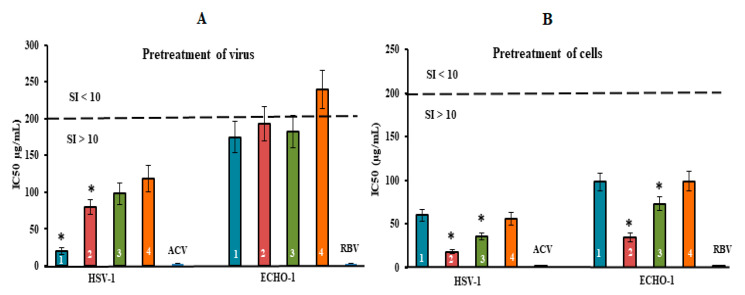
Inhibitory effect of compounds at the pre-entry stage of the viral replication cycle. (**A**) Virus (HSV-1 or ECHO-1) was preincubated with compounds for 1 h prior to infection of Vero cells; (**B**) Vero cells were treated with compounds for 1 h prior to infection with virus. CRGs (CRG1, CRG2, CRG3 and CRG4) are designated as 1, 2, 3 and 4, respectively; ACV—acyclovir and RBV—ribavirin. The dotted line represents a 50% inhibitory concentration (IC_50_) of 200 μg/mL, corresponding to a selective index (SI = CC_50_/IC_50_) of 10. * Significance of the differences between the parameters of CRG polysaccharides (CRG1, CRG2 and CRG3) compared to CRG oligosaccharide (CRG4) (*p* ≤ 0.05). The results include data from three experiments (mean ± SD).

**Figure 5:**
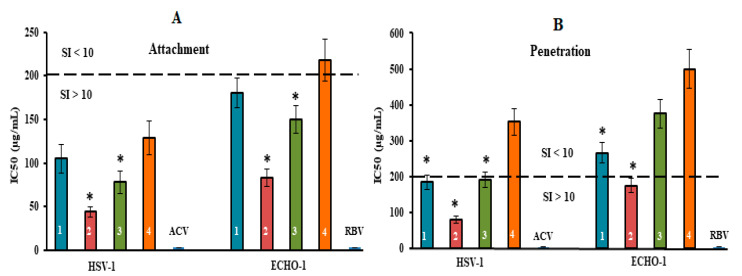
Inhibitory effect of compounds at the attachment and penetration stages of the viral replication cycle. (**A**) Vero cells were co-treated with the virus (HSV-1 or ECHO-1) and compounds at 4 °C; (**B**) cells were infected with the virus at 4 °C and then treated with the compounds at 37 °C. CRGs (CRG1, CRG2, CRG3 and CRG4) are designated as 1, 2, 3 and 4, respectively; ACV—acyclovir and RBV—ribavirin. The dotted line represents a 50% inhibitory concentration (IC50) of 200 μg/mL, corresponding to a selective index (SI = CC50/IC50) of 10. * Significance of the differences between the parameters of CRG polysaccharides (CRG1, CRG2 and CRG3) compared to CRG oligosaccharide (CRG4) (*p* ≤ 0.05). The results include data from three experiments (mean ± SD).

To determine the mode of antiviral action of the diverse structural types of CRGs against HSV-1 and ECHO-1 infections, time of addition experiments were conducted by a plaque reduction assay. To study the inhibitory effect of the tested compounds on the stage of virus infection, the compounds were added directly to the virus suspension (pre-treatment of the virus); cells were treated with compounds for 1 h before infection (pre-treatment of the cells); cells were co-treated with the virus and compounds at 4 °C (attachment); cells were infected with the virus at 4 °C and then treated with the compounds at 37 °C (penetration); and compounds were added 1 h after infection (treatment of infected cells). The obtained results were used for calculations of the IC_50_ and the SI for each of the compounds (Figure 4 and Figure 5 and Table S2). It is believed that compounds with SI ≥ 10 are promising for further investigation of the mechanism of antiviral action, including for in vivo studies [43].

The pre-treatment of enveloped DNA-containing virus (HSV-1) with CRGs (direct virucidal action) showed a moderate antiviral activity of CRG2, CRG3 and CRG4 (their mean IC_50_ of ~100 µg/mL and SI of ~20). At the same time, CRG1 exhibited higher virucidal activity compared to other CRGs: the IC_50_ of CRG1 was, on average, 2.0 times lower, and, accordingly, the SI was two times higher than those of other CRGs (*p* ≤ 0.05) (Figure 4A). In the case of non-enveloped RNA-containing ECHO-1 virus, the virucidal action of all four CRGs was modest (SI of ~10). Moreover, this method of application of acyclovir and ribavirin did not reveal their virucidal activity.

The treatment of Vero cells with CRGs before infection (preventive effect) revealed the highest antiviral activity of the tested compounds (Figure 4B and Table S2). Replication of HSV-1 was most effectively inhibited by CRG2 (IC_50_ = 18 μg/mL and SI = 111) and CRG3 (IC_50_ = 36 μg/mL and SI = 55). In the case of ECHO-1, the antiviral activity of the CRGs was moderate but as with HSV-1, CRG2 protected Vero cells against enterovirus infection, with an SI that was 2.5 times higher than the other CRGs (SI of ~22) (*p* ≤ 0.05), wherein acyclovir and ribavirin, with which Vero cells were pre-treated prior to infection, showed no preventive action against both viral infections.

Thus, the results of HSV-1 and ECHO-1 pre-treatment with CRGs and the results of pre-treatment of cells with CRGs revealed that these compounds affect the very early stages of the viral lifecycles, which are the attachment and penetration stages (Figure 5 and Table S2).

The tested compounds exhibited high anti-HSV-1 activity at the stage of virus attachment to cells (Figure 5A). CRG2 and CRG3 inhibited the binding of the virus to cells (SI = 45 and SI = 26, respectively) significantly more compared with CRG1 and CRG4 (mean SI of ~17) (*p* ≤ 0.05). In the attachment assay, during which Vero cells were co-treated with ECHO-1 and CRGs were tested at 4 °C, CRG1, CRG3 and CRG4 displayed a moderate inhibitory effect on the binding of the virus to cells (mean SI of ~11). At the same time, CRG2 significantly reduced the attachment of ECHO-1 to cells (SI = 24) compared to other CRGs (*p* ≤ 0.05).

The penetration assay, during which Vero cells were infected with virus at 4 °C and then treated with tested compounds at 37 °C, showed that the effect of CRG1, CRG3 and CRG4 on virus entry into cells was insignificant (SI ≤ 10) (Figure 5B), whereas treatment of the HSV-1-infected cells with CRG2 resulted in a significant reduction in plaque formation (SI = 25). Acyclovir and ribavirin did not affect the attachment and penetration stages of the corresponding viruses.

The application of CRGs (CRG1 and CRG3) after virus adsorption and penetration to cells (at 1 h post-infection) showed moderate replication inhibition against HSV-1 and ECHO-1 (average SI of 14) and slightly higher virus-inhibiting activity of CRG2 against these viruses (mean SI of ~23) (Table S2). Post-infection treatment of cells with CRGs-oligosaccharide (CRG4) and ribavirin had a weak effect on virus replication in contrast to acyclovir (SI of < 4 vs. 20,000).

### 2.4. Molecular Docking

**Figure 6:**
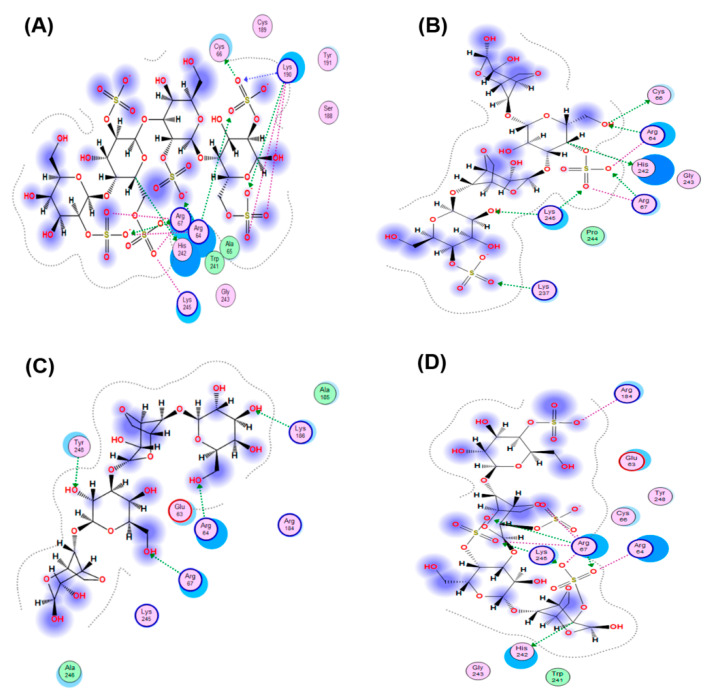
Molecular docking of CRGs into a potential binding site of the HSV-1 glycoprotein gD: (**A**) λ-CRG; (**B**) κ-CRG; (**C**) β–CRG; (**D**) ι-CRG. Abbreviations of aminoacids: alanine (Ala); arginine (Arg); aspartic acid (Asp); asparagine (Asn); glutamine (Gln); glycine (Gly); leucine (Leu); phenylalanine (Phe); serine (Ser); valine (Val).

**Figure 7:**
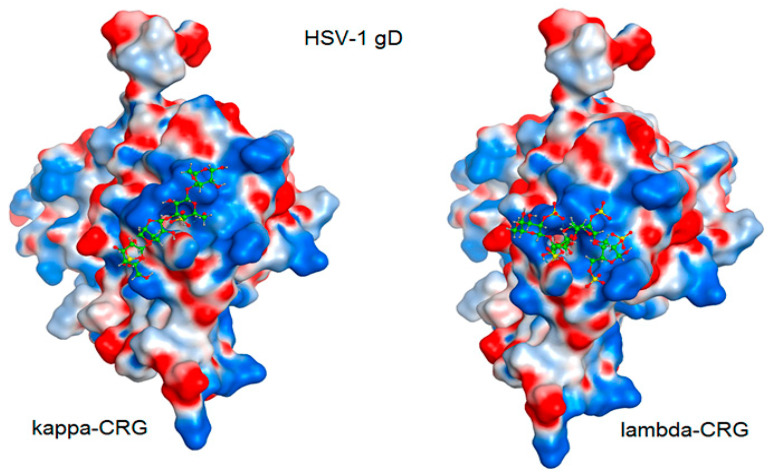
Molecular docking of CRG κ- and λ-tetrasaccharides with the putative binding site of the HSV-1 glycoprotein gD. The electrostatic potential of the molecular surface of the glycoprotein gD is shown in blue (electropositive) and red (electronegative) colors. Tetrasaccharide structures are shown in ball-and-stick form in green.

The interaction of the HSV-1 surface glycoprotein gD protein with the cellular receptors (nectin-1, 3-O-sulphated heparan sulphate (3-OS HS) and HVEM) triggers the adhesion of the virus to the cell and fusion with the cell membrane [44,45]. The putative binding site for 3-OS-HS of the glycoprotein gD HSV-1 was used for molecular docking of tetrasaccharides [46]. Virucidal compounds may affect gD protein and that inhibit the initial stages of HSV-1 infection. In this work, the interaction of CRG tetrasacharides with the HSV-1 surface glycoprotein gD was studied using molecular docking method. For modeling we used the tetrasaccharides of the corresponding CRGs—κ- and λ- which make up CRG1; CRG2 has of hybrid structure, which is built from units κ- and β-CRG and CRG3 from ι– and κ-CRG. This made it possible to calculate the energy of interaction of CRGs with the receptor gD and to assess the nature of the bonds involved in the binding. It was shown that the studied compounds can directly bind to gD (Figure 6), competing for binding sites of this protein with cellular receptor 3-OS HS, thereby reducing the HSV-1 activity to cells. Analysis of the contacts of the glycoprotein gD with CRG tetrasaccharides showed that sulfate groups of carrageenans form ionic and hydrogen bonds with arginine and lysine residues (Figure 7 and Table 2).

## 3. Discussion

The antiviral activity of sulphated polysaccharides has been reported to result from interference with early steps in the viral replication process, including virus adsorption. The mechanism of action can be attributed to an inhibitory effect on the initial attachment of the virus to the host cells and is thought to be mediated by interactions of sulphated polysaccharides with positively charged domains of the viral envelope glycoproteins involved in the attachment of the virus to heparan sulphate proteoglycans on the surface of the host cells [47].

Various investigators have reported the inhibitory effects of CRGs on the replication of many enveloped and some non-enveloped viruses. Their mechanisms of action are quite diverse, and they can act at different stages of the viral cycle, from preventing viral attachment to inhibiting the steps of intracellular replication. However, many researchers do not associate the manifestation of antiviral activity of CRGs with their structural features.

To identify possible relationships between the structure and antiviral activity of carrageenans, we investigated the effect of CRGs and oligosaccharide (CRG4) isolated from red algae of Pacific coast at different stages of the virus life cycle, such as HSV-1 and ECHO-1. Our data demonstrated the ability of CRGs to increase the resistance of Vero cells to virus infection (preventive effect), to directly affect virus particles (virucidal effect), and to inhibit the attachment and penetration of virus to cells. At the same time, tested CRGs more effectively inhibited the replication of the enveloped DNA virus HSV-1, compared with a moderate antiviral effect against non-enveloped RNA virus ECHO-1.

In addition, we investigated the antiviral effect of the tested CRGs, which is related to their chemical structure. CRG2 and CRG3 inhibited the binding of the virus to cells significantly more compared with CRG1 and CRG4. CRG2 and CRG3 exhibited high anti-HSV-1 activity at the stage of virus attachment to cells significantly more compared with CRG1 and CRG4. CRG2 significantly reduced the attachment of ECHO-1 to cells compared with the other CRGs. The differences in the activities of CRGs are thought to be largely associated with differences in their primary structure, including the content of 3,6-anhydrogalactose, the molar ratio of monosaccharide and sulphate group and the number and position of the sulphate group in the D-galactose residues. The polysaccharides CRG2 and CRG3 used in our work contain DA-units (3.6-anhydrogalactos). In CRG2 and CRG3, the 4-linked galactose residues, respectively DA adopt the 1C4-chair conformation. This conformation, which results from the 3,6-anhydro bridge, is crucial for the formation of the helical structure [48,49].

According to the data we received earlier, these CRGs form a three-dimensional network in solution due to intramolecular bonds between double helices of polysaccharide units, contain 3,6-anhydrogalactose [50]. Polysaccharides CRG2 and CRG3 have hybrid CRG structure but differ in the number of repeating units of CRG and the number of sulphated groups [51]. Thus, along with the κ-carrabiose units, the polymeric chain of CRG2 contains non-sulphated β-carrabiose units, while CRG3 contains strongly sulphated ι-carrabiose units. In the case of CRG2, which contains the least amount of sulphate groups, a denser network is formed. The formation of such network on the cell surface probably inhibits the adsorption of the virus, thus providing the greatest virus-inhibiting effect or attachment. CRG2 has the largest molecular weight (413 kDa), and the antiviral activity of sulphated polysaccharides is reported to increase with the molecular weight as well as with the sulphate content [47,52]. Moreover, CRG2 exhibits good mucoadhesive properties, as we have shown [53] which ensures its high adhesion to cells and prevents virus attachment. Similar to our results, ι/ν/κ-CRGs that contained DA-units were found to inhibit the adsorption of HSV to cells and protected mice from vaginal infection with HSV-2 when administered immediately prior to infection [54]. Unlike CRG2 and CRG3, CRG1 consists of κ- and λ-CRGs. λ-CRG does not form gels. This has been ascribed to the lack of DA residues, which are able to adopt a conformation that favor’s a helicoidal secondary structure required for gelation. The D2S,6S residues of λ-CRG, which lack the 3,6-anhydro bridge, adopt the 4C1–chair conformation [36,48,49,55]. In the conformation of the coil characteristic of λ-CRG, polymer molecules are highly hydrated intermolecular interactions are point-like in nature and, therefore, weak to ensure stable intermolecular association.

At the same time, CRG1 exhibited higher virucidal activity compared to the other CRGs. The polymer chains of λ-CRG are in the conformation of a chaotic coil, which ensures their flexibility. Such a structure of the polysaccharide probably allows CRG1, when directly exposed to the virus, to interact more effectively with some glycoproteins of the viral envelope, which are necessary for the adsorption of HSV-1 to cells. Moreover, CRG1 contains more sulphate group than other CRGs.

The molecular docking was used to study the interaction of CRGs with the HSV-1 surface glycoprotein, gD. It was found that the structure of tetrasaccharides affects the Docking score, binding energy value, and the number of ionic and hydrogen bonds (Table 2). λ-CRG the most sulfated tetrasaccharide, binds to glycoprotein gD more strongly than other CRG tetrasaccharides and this correlates with a stronger virucidal effect of CRG1, which contains λ-CRG, on HSV-1 (Figure 4A).

In our opinion, the presence in the CRG1 λ-CRG determines its greater virucidal effect in comparison with CRG2, which consistent with studies by other authors. For example, λ-CRGs and the partially cyclized μ/ν-CRG were the most potent inhibitors of herpes viruses (including acyclovir-resistant variants and clinical isolates) against both serotypes. Antiherpetic activity was directly correlated to the amount of α-d-galactose 2,6-disulfate residues in the natural CRGs [56]. In a similar study, the λ-CRGs from *G. skottsbergii* provided protection against HSV-2 when administered prior to infection and its activity was superior to μ/ν-CRG, suggesting that the virucidal activity of λ-CRGs may play an important role [15]. Damonte, et al. [47] showed that the direct virucidal actions of carrageenan may be due to the formation of a stable virion–CRG complex where binding is not reversible. Hence, the sites on the viral envelope required for virus attachment to host cells are occupied by the sulphated polysaccharide, which renders the virus unable to complete the subsequent infection process. In a similar study, the λ-CRG (1T1) from *G. skottsbergii* provided protection against HSV-2 when administered prior to infection and its activity was superior to μ/ν-CRG suggesting that the virucidal activity of λ-CRGs may play an important role [15]. In additional, significant virucidal activity of CRGs against the enveloped HSV-1 virus should also be noted

The study of the effect of CRGs at the stage of viral infection has shown the dependence of antiviral activity on the structural features of these polysaccharides.

## 4. Materials and Methods

### 4.1. Virus and Cell Culture

The following viruses were used for the study. HSV-1 strain L2 was obtained from N.F. Gamaleya Federal Research Centre for Epidemiology and Microbiology, Moscow, Russia. The strain IP91 of ECHO-1 enterovirus was obtained from the Chumakov Federal Scientific Center for Research and Development of Immune and Biological Products (Moscow, Russia).

Determination of the cytotoxicity and antiviral activity of the compounds was carried out on a Vero cell culture (kidney epithelial cells of the African green monkey *Chlorocebus sp*.) obtained from N.F. Gamaleya Federal Research Centre for Epidemiology and Microbiology, Moscow, Russia. 

HSV-1 and ECHO-1 were grown in Vero cells using Dulbecco’s Modified Eagle’s Medium (DMEM, Biolot, St. Petersburg, Russia) supplemented with 10% fetal bovine serum (FBS, Biolot, St. Petersburg, Russia) and 100 U/mL of gentamycin (Dalkhimpharm, Khabarovsk, Russia) at 37 °C in a CO_2_ incubator. In the maintenance medium, the FBS concentration was decreased to 1%.

### 4.2. Algal Material

Red algae from family Gigartinaceae (*Chondrus armatus*), Tichocarpaceae (*Tichocarpus crinitus*), and Phyllophoraceae (*Ahnfeltiopsis flabelliformis*) were harvested at Peter the Great Bay, Sea of Japan, and identified based on morphological and anatomical characteristics by Prof. E. Titlyanov and T. Titlyanova (National Scientific Center of Marine Biology, Far-Eastern Branch of the Russian Academy of Sciences) using an transmission electron microscope. The selected seaweeds *C. armatus* and *T. crinitus* are gametophytes lacking any reproductive organs, whereas *A. flabelliformis* is a cystocarpic plant. The algae were washed with tap water to remove excess salt. Bleaching of the seaweed was performed by maintaining the specimen in pure acetone for 3 days prior to being dried in the air.

### 4.3. Extraction of Carrageenans

Dried and milled algae (50 g) were suspended in hot water (1.5 L) and the polysaccharides were extracted three times at 80 °C for 3 h in boiling water. Hot extracts were combined, centrifuged at 4000 rpm^−1^ to remove residues of the cell wall, filtered through a Vivaflow200 membrane (Sartorius, Germany) with a pore size of 100 kDa, concentrated on a rotary evaporator, and the polysaccharides precipitated with a triple volume of 96% ethanol, as described previously [38]. The precipitates as the crude extracts were purified by redissolving in water and were concentrated, dialyzed, and freeze-dried. The unfractionated or the total polysaccharides Σ-CRGs were obtained, and one from *C. armatus* was used in the experiments (CRG1). Then the Σ-CRGs from *T. crinitus* were separated with 4% KCl into gelling (KCl-insoluble) and non-gelling (KCl-soluble) fractions as described previously [38]. The gelling fraction (KCl-insoluble fraction) was used in the work (CRG2). Σ-CRGs from *A. flabelliformis* were separated into gelling and non-gelling fractions with 4% CaCl2 according to [42] and gelling fractions (CRG3) were used. The structure of the gelling polysaccharide was established according to the published protocol [42].

### 4.4. Obtaining Oligosaccharides by Mild Acid Hydrolysis

A polysaccharide sample (100 mg) was dissolved in 50 mL of 0.1 N HCl and heated at 37 °C for 44 h. Hydrolysis was stopped by the addition of some drops of 0.1 N NH_4_OH solution to pH 8–-9. Then, oligosaccharides were precipitated with five volumes of 96% ethanol and centrifuged at 4000 rpm^−1^ for 30 min at 4 °C. The precipitate was collected and lyophilized (CRG4).

### 4.5. Analytical Methods

The monosaccharide composition was determined by total reductive hydrolysis [57,58]. Neutral monosaccharides were analyzed as alditol [58]. To determine the content of 3,6-anhydrogalactose, total reductive hydrolysis of the CRGs in 2 M trifluoroacetic acid (TFA) (100 °C, 4 h) with 4-methylmorpholinborane was carried out, and then, aldononitrile acetate derivatives [57] by gas-liquid chromatography (GLC) with a 6850 chromatograph (“Agilent”, Santa Clara, CA, USA) equipped with a HP-5MS capillary column (30 m × 0.25 mm, 5% phenyl methyl siloxane) and a flame ionization detector. The analyses were carried out at a temperature gradient program from 150 to 230 °C; the rate of temperature change was 3 °C/min. The sulphate ester content of the polysaccharide was determined by the turbidimetric method [59].

### 4.6. Molecular Weight Estimation of CRGs

The molecular masses of the polysaccharides were calculated by the Mark-Houwink equation, as follows: [η] = K × Ma, where [η] is the intrinsic viscosity and K and α are empirical constants for CRG at 25 °C in 0.1 M NaCl, according to the literature data for this polymer-solvent system [60]. The viscosity of the polysaccharide solution (1–−2 mg/mL in 0.1 M NaCl) was measured with a modified Ubbellohde viscometer (Design Bureau, Pushchino, Russia) with a capillary diameter of 0.3 mm at 25 °C, with the time of accuracy being within ±0.1 s. The intrinsic viscosity of the CRG sample was calculated by the extrapolation of the dependence ln (η)rel/C to infinite dilution using the least squares method.

The average molecular weight of low molecular weight derivatives of polysaccharide was determined by the reducing sugars method with ferricyanide [61].

### 4.7. Virological Methods

For cytotoxicity and antiviral activity determination, the tested compounds—CRG1, CRG2, CRG3 and CRG4—were diluted in DMEM. Acyclovir and ribavirin were used as reference compounds. Acyclovir^®^, freeze-dried powder for injections (GlaxoSmithKline Pharmaceuticals S.A., Poznan, Poland) used for herpes virus infections, was diluted in DMEM. A stock solution (10 mg/mL) of Ribavirin^®^ (Sigma-Aldrich, St. Louis, MO, USA) used for enterovirus infections, was dissolved in dimethyl sulfoxide (DMSO, Sigma-Aldrich, St. Louis, MO, USA) and stored at −20 °C. For the experiments, it was diluted with DMEM to a final concentration of 0.5% DMSO.

#### 4.7.1. Cytotoxicity of the CRGs

The cytotoxicity evaluation of the studied carrageenans was performed using the MTT assay, as previously described [62,63]. In brief, confluent Vero cells (1 × 10^4^ cells/well) in 96-well microplates were incubated with various concentrations of the tested compounds (1–2000 μg/mL) at 37 °C for 72 h (5% CO_2_); untreated cells were used as controls. MTT solution (methylthiazolyltetrazolium bromide, Sigma, St. Louis, MO, USA) was added to cells at a concentration of 5 mg/mL, following incubation for 2 h at 37 °C. Then, the MTT solution was removed, and isopropanol was added to dissolve the insoluble formazan crystals. The optical density was read at 540 nm (Labsystems Multiskan RC, Vantaa, Finland). Cytotoxicity was expressed as the 50% cytotoxic concentration (CC_50_) of the tested compound that reduced the viability of treated cells by 50% compared with control cells. Experiments were performed in triplicate and repeated three times.

#### 4.7.2. Antiviral Activity of the CRGs

The antiviral activity of the tested compounds against HSV-1 and ECHO-1 was evaluated using the plaque reduction assay in Vero cells [64,65]. Vero cell monolayers grown in 24-well plates (1 × 10^5^ cells/well) were infected with 100 PFU/mL of the corresponding virus (HSV-1, ECHO-1) and simultaneously treated with the tested compounds (100 µL/well in triplicate) of different concentrations (from 1 to 500 µg/mL) for 1 h at 37 °C. After virus absorption, the virus–compound mixture was removed; the cells were washed with phosphate-buffered saline (PBS), covered with the maintenance medium (DMEM) containing 1% carboxymethyl cellulose (CMC, ICN Biomedicals Inc., Aurora, OH, USA), and incubated for 72 h at 37 °C (5% CO_2_) until plaques formed. After incubation, the cells were fixed with cold ethanol for 20 min, stained with a solution of 0.5% crystal violet in 20% ethanol, and the viral plaques were then counted. The plaque inhibition rate was calculated according to the following formula [66]: plaque inhibition (%) = 100 − [(PT/PC) × 100], where PT and PC are the plaque number of compound-treated infected cells and the virus control (without compound), respectively. The IC_50_ of each compound was determined as the compound concentration that inhibited 50% of viral plaque formation, compared to the control. The SI was calculated as the ratio of CC_50_ to IC_50_ for each compound. Experiments were repeated three times.

#### 4.7.3. Time of Compound Addition Assay

The inhibitory effects of the tested compounds on HSV-1 and ECHO-1 lifecycle stages in Vero cells were evaluated by the plaque reduction assay as described in Section 4.7.2. Vero cells were grown in 24-well plates (1 × 10^5^ cells/well), an infectious dose of both HSV-1 and ECHO-1 was of 100 PFU/mL, and tested compounds were used at various concentrations (1–500 µg/mL). Some schemes of the CRGs and appropriate pharmaceutical (acyclovir and ribavirin) application were investigated; each was carried out in three independent replicates in triplicate. The plates were incubated for 72 h at 37 °C (5% CO_2_) until plaques formed. These schemes were as follows:

- Pre-treatment of virus with compounds. The virus was mixed with compounds in a ratio of 1:1 (*v*/*v*), pre-incubated for 1 h at 37 °C, and then the mixture was used to infect cellular monolayers. After viral adsorption for 1 h at 37 °C, the cells were washed with PBS to remove unabsorbed virus and incubated with maintenance medium (DMEM) containing 1% CMC.
- Pre-treatment of cells with compounds. A monolayer of cells was pre-treated with compounds for 1 h at 37 °C before infection. The cells were washed with PBS to remove the compounds and infected with the virus for 1 h at 37 °C. Then, unabsorbed virus was removed by washing with PBS, and the cells were incubated with DMEM with 1% CMC.
- The attachment assay. The monolayer of cells was pre-chilled at 4 °C for 1 h and then treated with a mixture of virus and compound (1:1). After incubation at 4 °C for 3 h, the compounds and unabsorbed virus were removed by washing with cold PBS and the cells were incubated with DMEM with 1% CMC.
- The penetration assay. The monolayer of cells, pre-chilled at 4 °C for 1 h, was infected with the virus and incubated at 4 °C for 3 h. The unbound virus was removed with cold PBS and the infected cells were treated with the compounds and incubated for 1 h at 37 °C. Then, the unpenetrated virus was inactivated with citrate buffer (pH 3.0) and the cells were washed with PBS, and incubated with DMEM with 1% CMC.
- Treatment of infected cells. The monolayer of cells was infected with the virus at 37 °C for 1 h, then washed and overlaid with DMEM with 1% CMC containing different concentrations of the tested compounds.

In all assays, after 72 h of incubation, the viral plaques were counted and then the IC_50_ and SI were calculated for each compound as described above.

### 4.8. Molecular Docking

The structures of kappa-, beta-, iota- and lambda-CRG tetrasacharides were obtained using the molecular editor of the MOE 2020.0901 program [Molecular Operating Environment (MOE), 2020.09; Chemical Computing Group ULC, 1010 Sherbrooke St. West, Suite #910, Montreal, QC, Canada, H3A 2R7, 2020]. For molecular docking, CRG tetrasacharides structures were used, which were solvated in the aqueous phase and optimised with the forcefield Amber10:EHT. The crystal structures of the complexes of the HSV-1 gD glycoprotein with the HSEA/M receptor (PDB ID 1JMA) were used as a target protein. The calculation of the electrostatic potential of the molecular surface of glycoprotein gD was carried out using the MOE 2019.01 program. Molecular docking of glycoprotein gD with the CRG tetrasacharides was performed using the Dock module of the MOE 2020.09 software. The structures of 30 complexes were calculated with Score London dG, and the 5 most energetically advantageous complexes were optimised with Score GBVI/WSA dG. Contact analysis was carried out using the Ligand Interaction module of the MOE program.

### 4.9. Statistical Analysis

Statistical processing of the data was performed using the Statistica 10.0 software (StatSoftInc., Tulsa, OK, USA). CC50 and IC50 were calculated by regression analysis of the dose–response curves. The results are presented as the mean ± standard deviation (SD). The differences between the parameters of the control and experimental groups were estimated using the Wilcoxon test. Differences were considered significant at *p* ≤ 0.05.

## 5. Conclusions

In this work, we investigated the antiviral activity of the sulfated polysaccharides CRGs isolated from red algae of the Pacific coast. The structure of CRGs has been confirmed by the methods of IR Fourier and NMR spectroscopy. CRGs differ in the presence of 3.6 anhydrogalactose, the degree of sulfation and the distribution of sulfate groups along the polymer chain. We have demonstrated for the first time the anti-HSV-1 and anti-ECHO-1 activity of CRGs and showed that it depends on the structure of CRGs. We found that CRGs with different effects significantly increased the resistance of Vero cells to virus infection (preventive effect), directly affected virus particles (virucidal effect), and inhibited the attachment and penetration of virus to cells. CRGs were more effective against HSV-1, mainly due to their ability to bind with the HSV-1 surface glycoprotein, gD, to prevent virus–cell interactions. Obtaining CRGs with different structures can be a basis of a successful strategy for the development of promising broad-spectrum antivirals.

## Figures and Tables

**Table 1:** Characteristic of CRGs from *C. armatus*, *T. crinitus*, and *A. flabelliformis*.

Source of CRG	Sample of CRGs	Structure of Disaccharide Repeating Unit		Molar Ratio AnGal: Gal:SO_4_^−2^	MW, kDa
		3-Linked	4-Linked		
ΣCRG κ + λ *C. armatus*	CRG κ CRG λ	G4S G2S	DA D2S,6S	1:15:1.8 1:18:12.5	185.0
*T. crinitus*	CRG2 κ/β-	G4S/G	DA/DA	1: 0.75:0.5	413.0
*A. flabelliformis*	CRG3 ι/κ-	G4S/G4S	DA2S/DA	1:1.8:2.9	307.0
	CRG3 ι/κ-OS	G4S/G4S	DA2S/DA	1:1.6:2.0	9.1

G—3-linked β-D-galactopyranose; D—4-linked α-D-galactopyranose; DA—4-linked 3,6-anhydro-α-D-galactopyranose; S—sulphate group; Gal—galactose; 3,6-AnGal—3,6-anhydrogalactose; SO_4_^−2^—sulphate group. Schematic representation of the different structures of disaccharide of the repeating units of carrageenans is presented in the supplementary materials.

**Table 2:** The binding energy of tetrasaccharides –CRGs and number of contacts in a complex with glycoprotein gD HSV-1.

CRG Units	E (kcal/mol)	Don	h-Acceptor	Ionic
β	−11.8	–	5	–
k	−43.4	2	5	5
i	−59.2	1	4	15
λ	−75.9	2	7	14

## Data Availability

The data presented in this study are available in this article and Supplementary Materials.

## Supplementary Materials

The following are available online at https://www.mdpi.com/article/10.3390/md20010060/s1, Scheme S1: Schematic representation of the different structures of disaccharide of the repeating units of carrageenans, Figure S1: ^1^H-NMR spectrum of κ/β-CRG from *T. crinitus*, Figure S2: ^13^C NMR spectrum of ι/κ-CRG from *A. flabelliformis*, Table S1: ^1^H and ^13^C-NMR chemical shifts of the signals κ/β-CRG from *T. crinitus*, Table S2: Antiviral activity of carrageenans.
