# Supplementary material for: Influence of the Structural Features of Carrageenans from Red Algae of the Far Eastern Seas on Their Antiviral Properties
Source: Mar Drugs. 2022 Jan 8;20(1):60. doi: 10.3390/md20010060 (PMC8779503; doi:10.3390/md20010060)
Supplement: Supplementary file 1 [file marinedrugs-20-00060-s001.zip › marinedrugs-1531129-supplementary.pdf]

Supplementary materials

Influence of the Structural Features of Carrageenans from Red Algae of the Far Eastern Seas on Their Antiviral Properties

Natalia V. Krylova [1,]*, Anna O. Kravchenko [2], Olga V. Iunikhina [1], Anastasia B. Pott [1], Galina N. Likhatskaya [2], Aleksandra V. Volod'ko [2], Tatyana S. Zaporozhets [1], Mikhail Y. Shchelkanov [1], Irina M. Yermak [2]*

ι/κ-CRG from *A. flabelliformis*

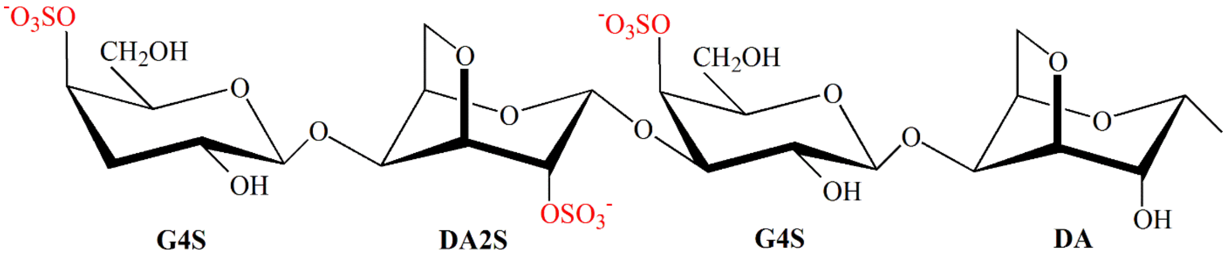

ι -unit κ -unit

κ/β-CRG from *T. crinitus*

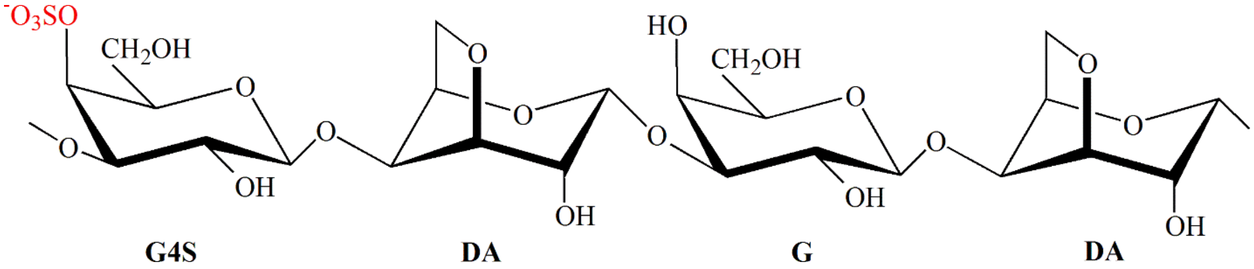

κ -unit β-unit

λ - CRG from *C. armatus*

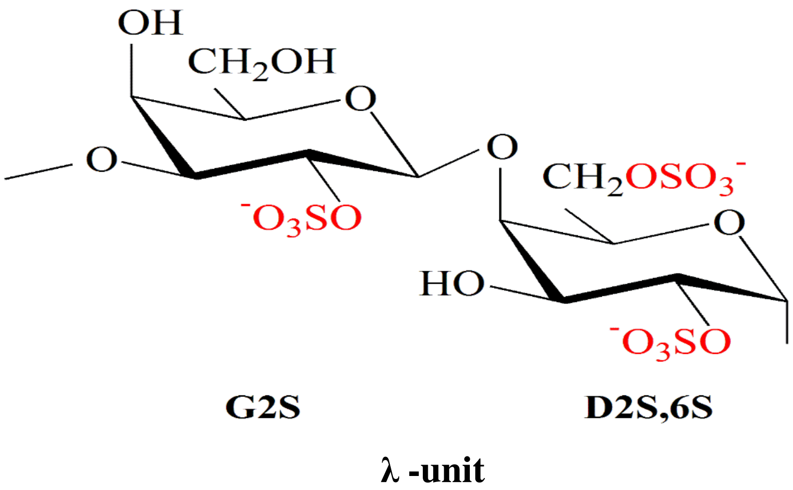

Scheme S1. Schematic representation of the different structures of disaccharide of the repeating units of carrageenans.

Table S1. ^{1}H and ^{13}C-NMR chemical shifts of the signals κ/β- CRG from *T. crinitus*

Type of CRG	units	$^{1}H/^{13}C$ chemical shift (ppm)					
		**H-1/C-1**	**H-2/C-2**	**H-3/C-3**	**H-4/C-4**	**H-5/C-5**	**H-6/C-6**
κ -	G4S	4.7/103.1	3.7/70.0	4.0/79.0	4.9/74.6	4.0/75.1	3.8/61.1
	DA	5.1/95.1	4.0/69.7	4.5/80.1	4.6/79.1	4.7/78.1	4.2/69.5
β -	G	4.6/102.7	3.6/70.1	3.9/80.8	4.1/66.1	3.7/75.2	3.8/61.1
	DA′	5.1/94.4	4.1/70.4	4.5/79.2	4.6/78.2	4.6/76.7	4.2/69.7

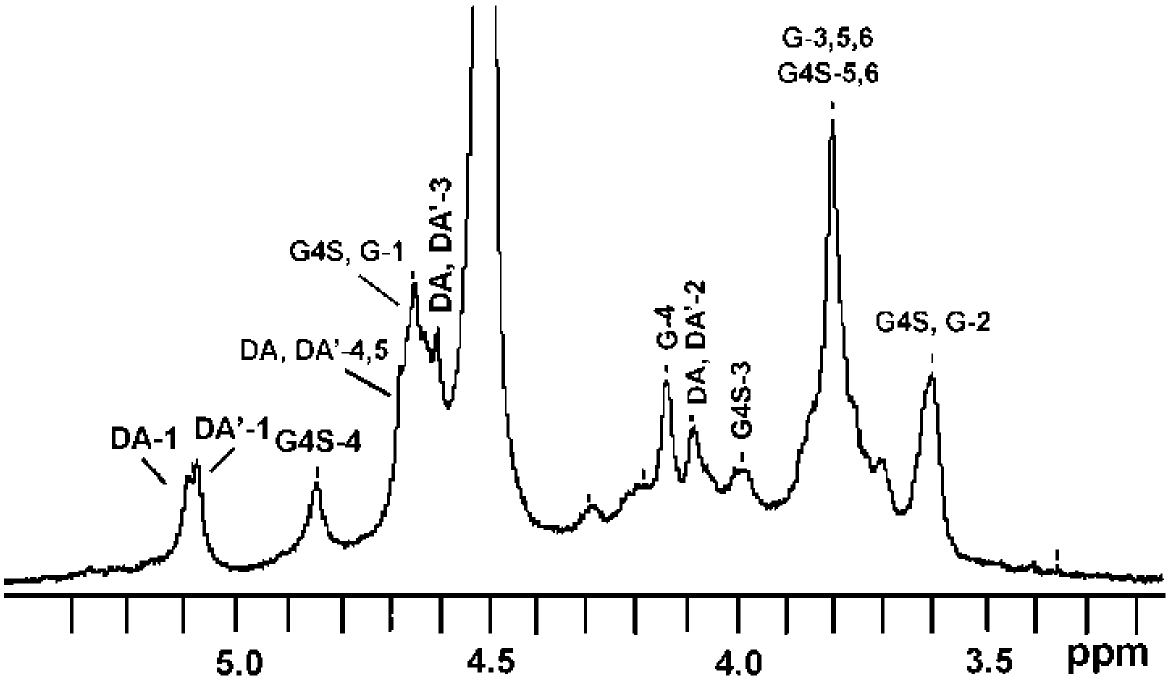

Figure S1. ^{1}H- NMR spectrum of κ/β- CRG from *T. crinitus*

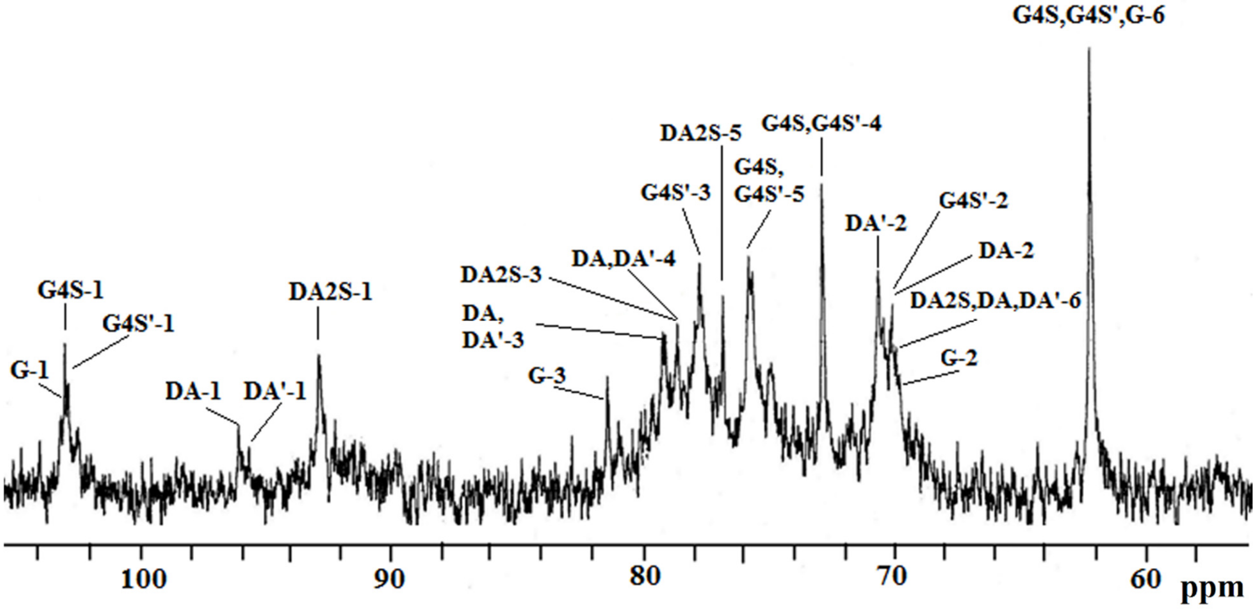

Figure S2. ^{13}C NMR spectrum of ι/κ-CRG from *A. flabelliformis*

Table S2. Antiviral activity of carrageenans

Viruses	Compounds	Pretreatment of Virus		Pretreatment of Cells		Attachment		Penetration		Treatment of Infected Cells	
		IC_{50} (μ/mL)	SI	IC_{50} (μ/mL)	SI	IC_{50} (μ/mL)	SI	IC_{50} (μ/mL)	SI	IC_{50} (μ/mL)	SI
**HSV-1**	CRG1	20 ± 4 *	100 ± 5	60 ± 7	33 ± 4	105 ± 16	19 ± 3	185 ± 20 *	11 ± 2	103 ± 14 *	19 ± 3
	CRG2	80 ± 10 *	25 ± 3	18 ± 3 *	111 ± 14	44 ± 6 *	45 ± 6	81 ± 10 *	25 ± 3	77 ± 11 *	26 ± 4
	CRG3	98 ± 15	20 ± 3	36 ± 5 *	55 ± 7	78 ± 13 *	26 ± 3	190 ± 21 *	10 ± 1	148 ± 19 *	14 ± 2
	CRG4	119 ± 18	17 ± 2	56 ± 7	36 ± 4	129 ± 19	15 ± 2	352 ± 38	6 ± 1	> 500	< 4
	ACV	NA		NA		NA		NA		0,1±0,01	>20.000
**ECHO-1**	CRG1	175 ± 26	11 ± 2	98 ± 10	20 ± 2	181 ± 17	11 ± 2	266 ± 29 *	7 ± 1	180 ± 23 *	11 ± 1
	CRG2	193 ± 27	10 ± 2	34 ± 5 *	59 ± 7	83 ± 10 *	24 ± 4	175 ± 19 *	11 ± 2	100 ± 14 *	20 ± 3
	CRG3	182 ± 25	11 ± 2	73 ± 8 *	27 ± 3	150 ± 16 *	13 ± 2	375 ± 52	5 ± 1	162 ± 21 *	12 ± 1
	CRG4	240 ± 31	8 ± 1	99 ± 11	20 ± 2	218 ± 24	9 ± 1	500 ± 75	4 ± 1	> 500	< 4
	RBV	NA		NA		NA		NA		> 500	< 4

Note: Values represent the means ± standard deviations of three or more independent experiments; CRG1 – CRG4 - various structural types of carrageenans. Acyclovir and ribavirin were used as reference compound; IC_{50}, concentration that inhibited 50% of viral plaque formation; SI, selectivity index (CC_{50} /IC_{50}). * Significance of the differences between the parameters of carrageenan polysaccharides (CRG1, CRG2 and CRG3) compared to carrageenan oligosaccharide (CRG4) ($p \leq 0.05$).
